# 4-Phenyl-1*H*-imidazole-2(3*H*)-thione

**DOI:** 10.1107/S1600536812020090

**Published:** 2012-05-12

**Authors:** Anita M. Owczarzak, Maciej Kubicki

**Affiliations:** a Department of Chemistry, Adam Mickiewicz University, Grunwaldzka 6, 60-780 Poznań, Poland

## Abstract

In the asymmetric unit of the title compound, C_9_H_8_N_2_S, there are four symmetry-independent mol­ecules (*Z*′ = 4). The geometrical features of these mol­ecules are quite similar: in the normal probability plots the *R* ^2^ correlation factors for bond lengths and angles are generally around 0.95. The twist angles between the imidazole and phenyl rings (which are planar within 3σ) range from 9.0 (6) to 13.1 (5)°. In the crystal, pairs of independent molecules are joined by linear N—H⋯S and weak C—H⋯S hydrogen bonds, forming infinite ribbons, of the type ∼*ABABAB*∼ and ∼*CDCDCD*∼, propagating along [110]. Second-order hydrogen-bonded *R*_2_^2^(8) rings are formed *via* inter­weaving infinite *C*_2_^2^(8) chains.

## Related literature

For related structures, see: Conde *et al.* (1977 ▶); Raper *et al.* (1984 ▶). For general background to thio­amides, see: Martindale (1982 ▶); Hussain *et al.* (1990 ▶); Buxeraud (1995 ▶). For normal probability plots, see: Abrahams & Keve (1971 ▶); *Inter­national Tables for X-ray Crystallography* (1974 ▶). For a description of the Cambridge Structural Database, see: Allen (2002 ▶). For graph-set notation, see: Bernstein *et al.* (1995 ▶); Etter *et al.* (1990 ▶).
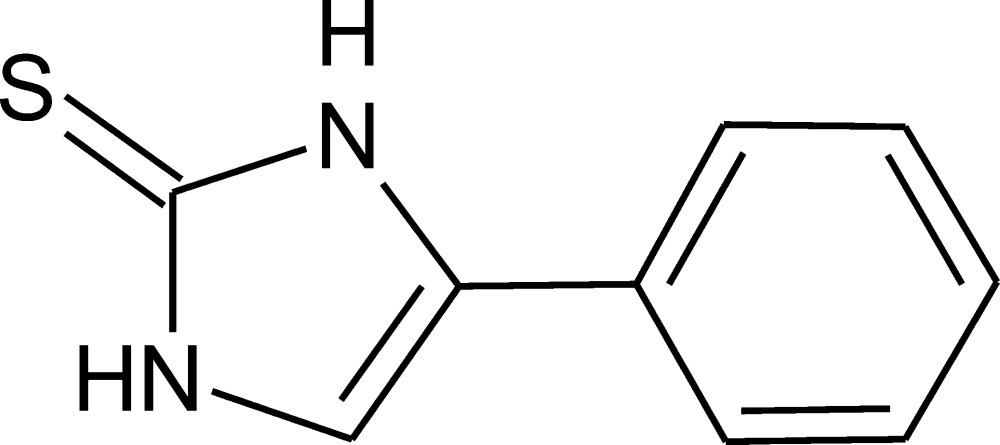


## Experimental

### 

#### Crystal data

C_9_H_8_N_2_S
*M* *_r_* = 176.23
Monoclinic, 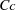

*a* = 11.7578 (5) Å
*b* = 11.8071 (5) Å
*c* = 25.1339 (18) Å
β = 91.858 (6)°
*V* = 3487.4 (3) Å^3^
*Z* = 16
Mo *K*α radiation
μ = 0.31 mm^−1^
*T* = 295 K
0.2 × 0.16 × 0.03 mm

#### Data collection

Agilent Xcalibur, Eos diffractometer
Absorption correction: multi-scan (*CrysAlis PRO*; Agilent, 2011 ▶) *T*_min_ = 0.928, *T*_max_ = 1.000
11682 measured reflections
5247 independent reflections
4168 reflections with *I* > 2σ(*I*)
*R*_int_ = 0.045

#### Refinement

*R*[*F* ^2^ > 2σ(*F* ^2^)] = 0.065
*wR*(*F* ^2^) = 0.165
*S* = 1.08
5247 reflections
433 parameters
2 restraints
H-atom parameters constrained
Δρ_max_ = 0.80 e Å^−3^
Δρ_min_ = −0.27 e Å^−3^
Absolute structure: Flack (1983 ▶), 1447 Friedel pairs
Flack parameter: 0.01 (13)

### 

Data collection: *CrysAlis PRO* (Agilent, 2011 ▶); cell refinement: *CrysAlis PRO*; data reduction: *CrysAlis PRO*; program(s) used to solve structure: *SIR92* (Altomare *et al.*, 1993 ▶); program(s) used to refine structure: *SHELXL97* (Sheldrick, 2008 ▶); molecular graphics: *XP* (Sheldrick, 2008 ▶) and *Mercury* (Macrae *et al.*, 2008 ▶); software used to prepare material for publication: *SHELXL97*.

## Supplementary Material

Crystal structure: contains datablock(s) I, global. DOI: 10.1107/S1600536812020090/nk2157sup1.cif

Structure factors: contains datablock(s) I. DOI: 10.1107/S1600536812020090/nk2157Isup2.hkl

Supplementary material file. DOI: 10.1107/S1600536812020090/nk2157Isup3.cml

Additional supplementary materials:  crystallographic information; 3D view; checkCIF report

## Figures and Tables

**Table 1:** Hydrogen-bond geometry (Å, °)

*D*—H⋯*A*	*D*—H	H⋯*A*	*D*⋯*A*	*D*—H⋯*A*
N1*A*—H1*A*⋯S2*B*	0.86	2.44	3.274 (6)	163
N3*A*—H3*A*⋯S2*B*^i^	0.86	2.50	3.350 (5)	172
C42*A*—H42*A*⋯S2*B*^i^	0.93	2.85	3.723 (9)	156
N1*B*—H1*B*⋯S2*A*^ii^	0.86	2.46	3.290 (5)	163
N3*B*—H3*B*⋯S2*A*	0.86	2.48	3.343 (5)	176
C42*B*—H42*B*⋯S2*A*	0.93	2.79	3.686 (9)	161
N1*C*—H1*C*⋯S2*D*	0.86	2.45	3.288 (5)	166
N3*C*—H3*C*⋯S2*D*^ii^	0.86	2.50	3.348 (5)	172
C42*C*—H42*C*⋯S2*D*^ii^	0.93	2.89	3.741 (8)	153
N1*D*—H1*D*⋯S2*C*^i^	0.86	2.43	3.271 (6)	166
N3*D*—H3*D*⋯S2*C*	0.86	2.50	3.353 (6)	172
C42*D*—H42*D*⋯S2*C*	0.93	2.85	3.766 (9)	169

Symmetry codes: (i) 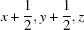; (ii) 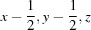.

**Fig. 1.**
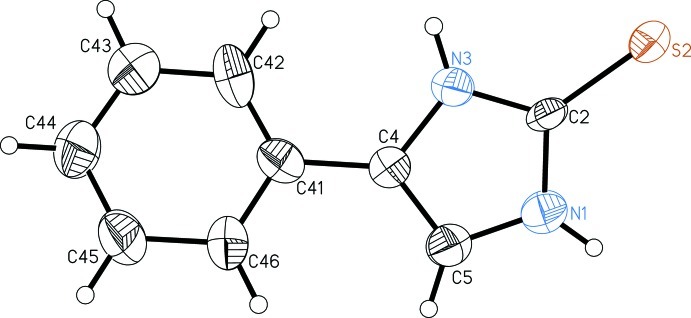
Anisotropic displacement ellipsoid representation of the molecule 1 A,The ellipsoids are drawn at the 50% probability level.

**Fig. 2.**
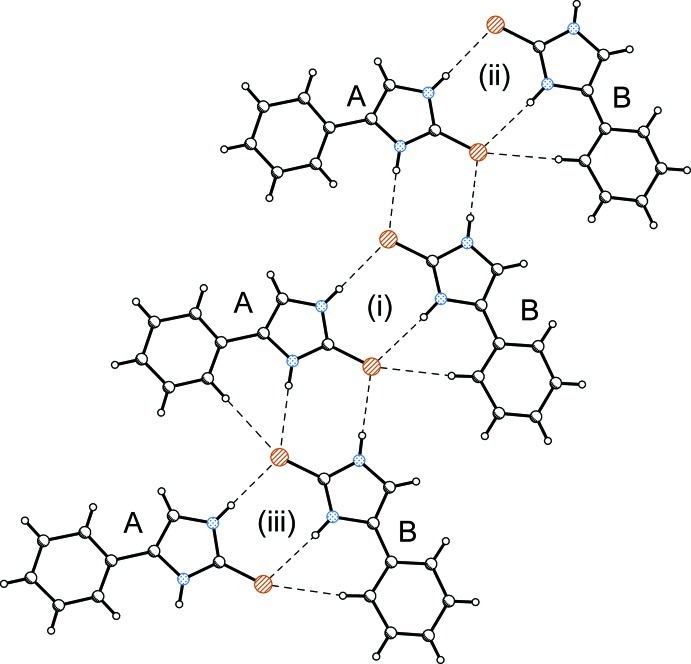
The hydrogen-bonded ribbon of molecules A and B (molecules C and D are joined into almost identical structure). Hydrogen bonds are shown as dashed lines, symmetry codes:; (i) -1/2 + x,-1/2 + y,z; (ii) 1/2 + x,1/2 + y,z..

**Fig. 3.**
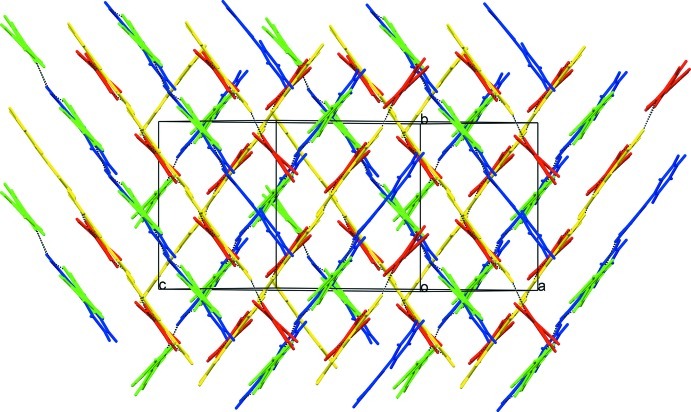
The crystal packing as seen approximately along c-direction, hydrogen bonds are drawn as dashed lines. Symmetry-independent molecules are shown with different colours.

## supplementary crystallographic information

### Comment

Heterocyclic thioamides are an important class of N, S-donor ligands, which display both hard and soft donor sites. They form a huge variety of coordination compounds and consequently have wide-ranging applications: for instance, as analytical reagents or metal corrosion inhibitors. They are also used as biologically active molecules (*e.g.* Hussain *et al.*, 1990, and references therein). Among the anti-thyroidal agents most widely used for the treatment of Graves' disease are some derivatives of imidazole-2-thiol as *N*-methylimidazoline-2-thione (Methimazole) as well as other thioamides, *e.g.* 3-methyl-2-thioxo-4-imidazoline-1-carboxylate (Carbimazole) and propylthiouracil (Martindale, 1982, Buxeraud, 1995). Here we present the crystal structure of simple thioamide, 4-phenyl-1,3-dihydro-2*H*-imidazole-2-thione (1, Scheme 1), which turned out to crystallize with *Z*'=4.

The Cambridge Structural Database (Allen, 2002) contains only a handful of 1,3-dihydroimidazole-2-thione derivatives. These are mainly S-metal complexes and few simple organic derivatives, for instance 1,3-dihydro-2*H*-imidazole-2-thione hydrate (Raper *et al.*, 1984) and 4-formyl-1,3-dihydro-2*H*-imidazole-2-thione (Conde *et al.*, 1977).

The asymmetric part of the unit cell of 1 contains four independent molecules, Fig. 1 shows one of them. These molecules are similar; the results of the normal probability plot analysis (International Tables for X-ray Crystallography, 1974; Abrahams & Keve, 1971) for bond lengths and angles show that there are no systematical differences between the molecules, and the actual differences are only of statistical nature: *R*^2^ correlation factors for bond lengths and angles are generally around 0.95.

Overall conformation of the molecules can be described here by the dihedral angles between two almost perfectly planar (within 3 s.u.'s) rings, imidazole and phenyl. These angles are relatively small - thanks partially at least to the lack of the sterical hindrance - and range from 9.0 (6)° for molecule B to 13.1 (5)° for molecule C. The bond length and angles are typical, with the C—S bond distance confirming its double-bond character, the mean value of this length is 1.694 (4) Å.

In the crystal structure the pairs of molecules A—B and C—D create identical but independent motifs. They are joined into infinite ribbons (along [110]) by means of relatively short and linear N—H···S hydrogen bonds (Table 1, Fig. 2), and additionally by weaker, secondary C—H···S hydrogen bonds. Using graph-set notation (Etter, *et al.*, 1990, Bernstein *et al.*, 1995), one can identify the second-order rings *R*^2^_2_(8) which are made by interweaving C^2^_2_(8) chains. These almost independent ribbons combine together to make the overall three-dimensional structure (Fig. 3).

### Experimental

The title compound was prepared by adding hydrochloric acid to acetonitrile solution of 4-phenyl-imidazole-2-thiol in molar ratio 1:1. After a few minutes colourless, thin crystals of 1, suitable for single-crystal X-ray analysis appeared and were filtered off.

### Refinement

Hydrogen atoms were put in the idealized positions, and refined as riding model. Their isotropic thermal parameters were set at 1.2 times *U*_eq_'s of appropriate carrier atoms.

### Crystal data

**Table:**

C_9_H_8_N_2_S	*F*(000) = 1472
*M**_r_* = 176.23	*D*_x_ = 1.343 Mg m^−^^3^
Monoclinic, *C**c*	Mo *K*α radiation, λ = 0.71073 Å
Hall symbol: C -2yc	Cell parameters from 4788 reflections
*a* = 11.7578 (5) Å	θ = 2.4–26.9°
*b* = 11.8071 (5) Å	µ = 0.31 mm^−^^1^
*c* = 25.1339 (18) Å	*T* = 295 K
β = 91.858 (6)°	Plate, yellow
*V* = 3487.4 (3) Å^3^	0.2 × 0.16 × 0.03 mm
*Z* = 16	

### Data collection

**Table:**

Xcalibur, Eos diffractometer	5247 independent reflections
Radiation source: Enhance (Mo) X-ray Source	4168 reflections with *I* > 2σ(*I*)
Graphite monochromator	*R*_int_ = 0.045
Detector resolution: 16.1544 pixels mm^-1^	θ_max_ = 27.0°, θ_min_ = 2.5°
ω–scan	*h* = −14→9
Absorption correction: multi-scan (*CrysAlis PRO*; Agilent, 2011)	*k* = −14→14
*T*_min_ = 0.928, *T*_max_ = 1.000	*l* = −30→30
11682 measured reflections	

### Refinement

**Table:**

Refinement on *F*^2^	Secondary atom site location: difference Fourier map
Least-squares matrix: full	Hydrogen site location: inferred from neighbouring sites
*R*[*F*^2^ > 2σ(*F*^2^)] = 0.065	H-atom parameters constrained
*wR*(*F*^2^) = 0.165	*w* = 1/[σ^2^(*F*_o_^2^) + (0.073*P*)^2^ + 5.346*P*] where *P* = (*F*_o_^2^ + 2*F*_c_^2^)/3
*S* = 1.08	(Δ/σ)_max_ = 0.001
5247 reflections	Δρ_max_ = 0.80 e Å^−^^3^
433 parameters	Δρ_min_ = −0.27 e Å^−^^3^
2 restraints	Absolute structure: Flack (1983), 1447 Friedel pairs
Primary atom site location: structure-invariant direct methods	Flack parameter: 0.01 (13)

### Special details

**Table:**

Geometry. All s.u.'s (except the s.u. in the dihedral angle between two l.s. planes) are estimated using the full covariance matrix. The cell s.u.'s are taken into account individually in the estimation of s.u.'s in distances, angles and torsion angles; correlations between s.u.'s in cell parameters are only used when they are defined by crystal symmetry. An approximate (isotropic) treatment of cell s.u.'s is used for estimating s.u.'s involving l.s. planes.
Refinement. Refinement of *F*^2^ against ALL reflections. The weighted *R*-factor *wR* and goodness of fit *S* are based on *F*^2^, conventional *R*-factors *R* are based on *F*, with *F* set to zero for negative *F*^2^. The threshold expression of *F*^2^ > 2σ(*F*^2^) is used only for calculating *R*-factors(gt) *etc*. and is not relevant to the choice of reflections for refinement. *R*-factors based on *F*^2^ are statistically about twice as large as those based on *F*, and *R*- factors based on ALL data will be even larger.

### Fractional atomic coordinates and isotropic or equivalent isotropic displacement parameters (Å^2^)

**Table:**

	*x*	*y*	*z*	*U*_iso_*/*U*_eq_	
N1A	0.4989 (5)	0.4546 (4)	0.8218 (2)	0.0444 (12)	
H1A	0.4424	0.4129	0.8115	0.053*	
C2A	0.5332 (5)	0.5485 (5)	0.7969 (3)	0.0345 (13)	
S2A	0.47435 (12)	0.60428 (14)	0.74045 (7)	0.0458 (4)	
N3A	0.6236 (4)	0.5869 (4)	0.8257 (2)	0.0371 (11)	
H3A	0.6619	0.6464	0.8179	0.045*	
C4A	0.6471 (5)	0.5183 (5)	0.8695 (2)	0.0360 (13)	
C41A	0.7430 (6)	0.5375 (5)	0.9085 (3)	0.0411 (15)	
C42A	0.8265 (8)	0.6105 (8)	0.8996 (4)	0.070 (3)	
H42A	0.8229	0.6528	0.8684	0.084*	
C43A	0.9180 (9)	0.6267 (9)	0.9345 (4)	0.079 (3)	
H43A	0.9745	0.6785	0.9263	0.095*	
C44A	0.9258 (7)	0.5679 (8)	0.9802 (4)	0.067 (2)	
H44A	0.9857	0.5797	1.0046	0.080*	
C45A	0.8436 (9)	0.4911 (8)	0.9894 (4)	0.072 (3)	
H45A	0.8495	0.4460	1.0197	0.086*	
C46A	0.7474 (7)	0.4777 (7)	0.9534 (3)	0.0558 (19)	
H46A	0.6890	0.4280	0.9613	0.067*	
C5A	0.5680 (6)	0.4349 (5)	0.8665 (3)	0.0428 (14)	
H5A	0.5613	0.3750	0.8902	0.051*	
N1B	0.0966 (4)	0.3495 (5)	0.7179 (2)	0.0435 (13)	
H1B	0.0559	0.2940	0.7286	0.052*	
C2B	0.1929 (5)	0.3860 (5)	0.7428 (2)	0.0378 (14)	
S2B	0.25122 (12)	0.33365 (14)	0.79993 (7)	0.0466 (4)	
N3B	0.2285 (4)	0.4711 (4)	0.7131 (2)	0.0358 (11)	
H3B	0.2896	0.5091	0.7199	0.043*	
C4B	0.1546 (5)	0.4916 (5)	0.6695 (2)	0.0348 (13)	
C41B	0.1702 (6)	0.5816 (6)	0.6307 (3)	0.0422 (15)	
C42B	0.2505 (8)	0.6634 (9)	0.6382 (4)	0.076 (3)	
H42B	0.2988	0.6638	0.6682	0.091*	
C43B	0.2579 (9)	0.7492 (9)	0.5979 (5)	0.096 (4)	
H43B	0.3120	0.8059	0.6032	0.116*	
C44B	0.1938 (8)	0.7536 (9)	0.5539 (4)	0.062 (2)	
H44B	0.2034	0.8092	0.5283	0.075*	
C45B	0.1127 (9)	0.6719 (8)	0.5484 (4)	0.076 (3)	
H45B	0.0639	0.6736	0.5186	0.091*	
C46B	0.0997 (8)	0.5848 (7)	0.5859 (3)	0.067 (2)	
H46B	0.0439	0.5298	0.5805	0.081*	
C5B	0.0719 (6)	0.4130 (5)	0.6731 (3)	0.0442 (15)	
H5B	0.0098	0.4034	0.6496	0.053*	
N1C	0.5331 (4)	0.2879 (4)	0.7138 (2)	0.0469 (13)	
H1C	0.5905	0.3287	0.7238	0.056*	
C2C	0.4983 (5)	0.1952 (5)	0.7392 (3)	0.0364 (13)	
S2C	0.55718 (13)	0.14044 (14)	0.79617 (7)	0.0481 (4)	
N3C	0.4080 (4)	0.1579 (4)	0.7113 (2)	0.0368 (11)	
H3C	0.3689	0.0991	0.7192	0.044*	
C4C	0.3853 (5)	0.2275 (5)	0.6673 (2)	0.0376 (14)	
C41C	0.2895 (5)	0.2125 (6)	0.6298 (3)	0.0425 (15)	
C42C	0.1987 (7)	0.1387 (7)	0.6398 (3)	0.053 (2)	
H42C	0.1989	0.0963	0.6710	0.064*	
C43C	0.1101 (8)	0.1297 (8)	0.6034 (4)	0.074 (3)	
H43C	0.0504	0.0803	0.6099	0.089*	
C44C	0.1083 (9)	0.1948 (7)	0.5560 (4)	0.070 (3)	
H44C	0.0477	0.1890	0.5314	0.084*	
C45C	0.1965 (7)	0.2658 (8)	0.5470 (3)	0.0518 (19)	
H45C	0.1962	0.3091	0.5161	0.062*	
C46C	0.2816 (7)	0.2737 (7)	0.5813 (3)	0.055 (2)	
H46C	0.3408	0.3226	0.5735	0.066*	
C5C	0.4655 (6)	0.3088 (6)	0.6699 (3)	0.0482 (16)	
H5C	0.4733	0.3683	0.6461	0.058*	
N1D	0.9343 (5)	0.3980 (5)	0.8200 (2)	0.0492 (14)	
H1D	0.9739	0.4548	0.8099	0.059*	
C2D	0.8402 (5)	0.3599 (5)	0.7950 (3)	0.0371 (13)	
S2D	0.77977 (13)	0.41209 (14)	0.73786 (7)	0.0503 (4)	
N3D	0.8053 (4)	0.2707 (4)	0.8242 (2)	0.0398 (12)	
H3D	0.7458	0.2307	0.8168	0.048*	
C4D	0.8767 (5)	0.2527 (5)	0.8668 (2)	0.0381 (14)	
C41D	0.8657 (6)	0.1604 (6)	0.9059 (3)	0.0421 (15)	
C42D	0.7813 (7)	0.0797 (7)	0.9028 (3)	0.061 (2)	
H42D	0.7277	0.0843	0.8748	0.073*	
C43D	0.7718 (9)	−0.0058 (9)	0.9380 (4)	0.084 (3)	
H43D	0.7117	−0.0570	0.9356	0.101*	
C44D	0.8559 (8)	−0.0141 (8)	0.9782 (4)	0.064 (2)	
H44D	0.8535	−0.0735	1.0024	0.077*	
C45D	0.9391 (7)	0.0611 (7)	0.9825 (3)	0.054 (2)	
H45D	0.9929	0.0559	1.0103	0.065*	
C46D	0.9459 (7)	0.1450 (7)	0.9465 (3)	0.0522 (19)	
H46D	1.0072	0.1947	0.9491	0.063*	
C5D	0.9591 (6)	0.3327 (6)	0.8646 (3)	0.0519 (17)	
H5D	1.0205	0.3419	0.8884	0.062*	

### Atomic displacement parameters (Å^2^)

**Table:**

	*U*^11^	*U*^22^	*U*^33^	*U*^12^	*U*^13^	*U*^23^
N1A	0.047 (3)	0.034 (3)	0.051 (3)	−0.008 (2)	−0.005 (3)	0.003 (2)
C2A	0.026 (3)	0.029 (3)	0.049 (3)	−0.008 (2)	0.001 (3)	0.009 (2)
S2A	0.0409 (9)	0.0470 (9)	0.0487 (9)	−0.0178 (7)	−0.0117 (7)	0.0096 (7)
N3A	0.036 (3)	0.036 (3)	0.040 (3)	−0.001 (2)	−0.001 (2)	0.009 (2)
C4A	0.036 (3)	0.035 (3)	0.037 (3)	−0.003 (3)	−0.001 (2)	0.003 (2)
C41A	0.044 (4)	0.040 (3)	0.039 (4)	0.006 (3)	0.002 (3)	0.006 (3)
C42A	0.062 (6)	0.087 (6)	0.059 (5)	0.002 (5)	−0.031 (4)	0.014 (5)
C43A	0.076 (6)	0.090 (6)	0.070 (6)	−0.033 (5)	−0.015 (5)	0.023 (5)
C44A	0.042 (4)	0.090 (6)	0.067 (5)	−0.015 (4)	−0.016 (4)	0.011 (5)
C45A	0.089 (7)	0.064 (5)	0.061 (5)	−0.012 (5)	−0.029 (5)	0.020 (4)
C46A	0.046 (4)	0.071 (5)	0.049 (4)	−0.009 (4)	−0.014 (3)	0.007 (4)
C5A	0.045 (4)	0.043 (3)	0.040 (3)	−0.010 (3)	0.000 (3)	0.002 (3)
N1B	0.035 (3)	0.043 (3)	0.052 (3)	−0.021 (2)	−0.004 (2)	0.002 (2)
C2B	0.039 (3)	0.031 (3)	0.044 (3)	0.002 (3)	0.003 (3)	0.000 (3)
S2B	0.0386 (9)	0.0488 (9)	0.0516 (9)	−0.0184 (7)	−0.0101 (7)	0.0157 (7)
N3B	0.023 (2)	0.039 (3)	0.045 (3)	−0.011 (2)	0.000 (2)	−0.005 (2)
C4B	0.035 (3)	0.039 (3)	0.031 (3)	0.002 (3)	−0.002 (2)	0.002 (2)
C41B	0.037 (4)	0.045 (4)	0.044 (4)	0.000 (3)	−0.005 (3)	−0.001 (3)
C42B	0.080 (7)	0.088 (6)	0.058 (5)	−0.014 (5)	−0.036 (5)	0.041 (5)
C43B	0.082 (7)	0.078 (6)	0.128 (10)	−0.036 (5)	−0.018 (7)	0.038 (6)
C44B	0.062 (5)	0.072 (6)	0.052 (5)	0.009 (4)	−0.001 (4)	0.022 (4)
C45B	0.107 (8)	0.077 (6)	0.042 (4)	−0.004 (6)	−0.030 (5)	0.024 (4)
C46B	0.082 (6)	0.066 (5)	0.052 (5)	−0.011 (4)	−0.013 (4)	0.013 (4)
C5B	0.042 (4)	0.043 (4)	0.047 (4)	−0.011 (3)	−0.011 (3)	0.001 (3)
N1C	0.039 (3)	0.042 (3)	0.059 (4)	−0.018 (2)	−0.002 (3)	0.005 (3)
C2C	0.031 (3)	0.035 (3)	0.042 (3)	−0.006 (2)	−0.003 (3)	−0.007 (3)
S2C	0.0442 (10)	0.0442 (9)	0.0550 (10)	−0.0178 (8)	−0.0112 (7)	0.0072 (8)
N3C	0.029 (3)	0.035 (2)	0.046 (3)	−0.014 (2)	0.002 (2)	−0.007 (2)
C4C	0.036 (3)	0.036 (3)	0.041 (3)	0.001 (3)	0.005 (3)	−0.003 (3)
C41C	0.033 (3)	0.049 (4)	0.046 (4)	−0.009 (3)	−0.006 (3)	−0.007 (3)
C42C	0.052 (5)	0.057 (5)	0.051 (4)	−0.017 (3)	0.000 (4)	0.020 (4)
C43C	0.052 (5)	0.074 (6)	0.094 (7)	−0.024 (4)	−0.024 (5)	0.016 (5)
C44C	0.093 (7)	0.059 (5)	0.055 (5)	−0.005 (5)	−0.038 (5)	0.008 (4)
C45C	0.047 (4)	0.069 (5)	0.039 (4)	−0.008 (4)	0.001 (3)	0.005 (3)
C46C	0.063 (5)	0.051 (4)	0.052 (4)	−0.006 (3)	0.010 (4)	0.016 (3)
C5C	0.050 (4)	0.046 (4)	0.049 (4)	−0.009 (3)	−0.004 (3)	0.012 (3)
N1D	0.043 (3)	0.049 (3)	0.054 (4)	−0.008 (3)	−0.004 (3)	0.008 (3)
C2D	0.022 (3)	0.040 (3)	0.050 (3)	−0.011 (2)	0.005 (2)	−0.006 (3)
S2D	0.0433 (10)	0.0495 (10)	0.0578 (10)	−0.0173 (7)	−0.0051 (8)	0.0106 (8)
N3D	0.039 (3)	0.034 (3)	0.046 (3)	−0.006 (2)	0.004 (2)	0.004 (2)
C4D	0.033 (3)	0.040 (3)	0.042 (3)	0.000 (3)	0.002 (3)	−0.009 (3)
C41D	0.043 (4)	0.039 (3)	0.044 (4)	−0.001 (3)	0.006 (3)	−0.003 (3)
C42D	0.061 (5)	0.059 (5)	0.062 (5)	−0.024 (4)	−0.022 (4)	0.017 (4)
C43D	0.085 (7)	0.091 (6)	0.074 (6)	−0.043 (5)	−0.034 (5)	0.039 (5)
C44D	0.073 (6)	0.055 (5)	0.064 (5)	0.003 (4)	0.010 (4)	0.019 (4)
C45D	0.036 (4)	0.075 (5)	0.050 (4)	0.004 (4)	0.005 (3)	−0.006 (4)
C46D	0.043 (4)	0.059 (4)	0.055 (4)	−0.012 (3)	−0.004 (3)	0.000 (3)
C5D	0.045 (4)	0.054 (4)	0.055 (4)	−0.012 (3)	−0.011 (3)	0.006 (3)

### Geometric parameters (Å, º)

**Table:**

N1A—C2A	1.342 (7)	N1C—C2C	1.338 (8)
N1A—C5A	1.385 (8)	N1C—C5C	1.361 (9)
N1A—H1A	0.8600	N1C—H1C	0.8600
C2A—N3A	1.345 (8)	C2C—N3C	1.329 (8)
C2A—S2A	1.691 (6)	C2C—S2C	1.698 (7)
N3A—C4A	1.388 (8)	N3C—C4C	1.397 (8)
N3A—H3A	0.8600	N3C—H3C	0.8600
C4A—C5A	1.355 (9)	C4C—C5C	1.345 (9)
C4A—C41A	1.487 (9)	C4C—C41C	1.456 (9)
C41A—C46A	1.331 (10)	C41C—C42C	1.407 (9)
C41A—C42A	1.331 (11)	C41C—C46C	1.416 (10)
C42A—C43A	1.378 (13)	C42C—C43C	1.368 (13)
C42A—H42A	0.9300	C42C—H42C	0.9300
C43A—C44A	1.343 (14)	C43C—C44C	1.419 (13)
C43A—H43A	0.9300	C43C—H43C	0.9300
C44A—C45A	1.351 (12)	C44C—C45C	1.358 (12)
C44A—H44A	0.9300	C44C—H44C	0.9300
C45A—C46A	1.434 (12)	C45C—C46C	1.303 (12)
C45A—H45A	0.9300	C45C—H45C	0.9300
C46A—H46A	0.9300	C46C—H46C	0.9300
C5A—H5A	0.9300	C5C—H5C	0.9300
N1B—C2B	1.346 (8)	N1D—C2D	1.333 (8)
N1B—C5B	1.377 (8)	N1D—C5D	1.383 (9)
N1B—H1B	0.8600	N1D—H1D	0.8600
C2B—N3B	1.328 (8)	C2D—N3D	1.355 (8)
C2B—S2B	1.688 (6)	C2D—S2D	1.697 (7)
N3B—C4B	1.397 (8)	N3D—C4D	1.355 (8)
N3B—H3B	0.8600	N3D—H3D	0.8600
C4B—C5B	1.349 (9)	C4D—C5D	1.355 (9)
C4B—C41B	1.458 (9)	C4D—C41D	1.476 (9)
C41B—C42B	1.359 (12)	C41D—C42D	1.377 (10)
C41B—C46B	1.377 (11)	C41D—C46D	1.378 (11)
C42B—C43B	1.437 (13)	C42D—C43D	1.350 (12)
C42B—H42B	0.9300	C42D—H42D	0.9300
C43B—C44B	1.318 (14)	C43D—C44D	1.393 (13)
C43B—H43B	0.9300	C43D—H43D	0.9300
C44B—C45B	1.360 (13)	C44D—C45D	1.323 (12)
C44B—H44B	0.9300	C44D—H44D	0.9300
C45B—C46B	1.407 (12)	C45D—C46D	1.345 (12)
C45B—H45B	0.9300	C45D—H45D	0.9300
C46B—H46B	0.9300	C46D—H46D	0.9300
C5B—H5B	0.9300	C5D—H5D	0.9300
			
C2A—N1A—C5A	109.9 (5)	C2C—N1C—C5C	110.9 (5)
C2A—N1A—H1A	125.0	C2C—N1C—H1C	124.6
C5A—N1A—H1A	125.0	C5C—N1C—H1C	124.6
N1A—C2A—N3A	105.7 (5)	N3C—C2C—N1C	105.7 (6)
N1A—C2A—S2A	126.3 (5)	N3C—C2C—S2C	128.0 (5)
N3A—C2A—S2A	127.9 (4)	N1C—C2C—S2C	126.3 (5)
C2A—N3A—C4A	111.4 (5)	C2C—N3C—C4C	110.6 (5)
C2A—N3A—H3A	124.3	C2C—N3C—H3C	124.7
C4A—N3A—H3A	124.3	C4C—N3C—H3C	124.7
C5A—C4A—N3A	105.1 (6)	C5C—C4C—N3C	105.4 (6)
C5A—C4A—C41A	130.7 (6)	C5C—C4C—C41C	130.1 (6)
N3A—C4A—C41A	124.2 (6)	N3C—C4C—C41C	124.4 (6)
C46A—C41A—C42A	118.5 (8)	C42C—C41C—C46C	116.1 (7)
C46A—C41A—C4A	119.1 (7)	C42C—C41C—C4C	122.4 (7)
C42A—C41A—C4A	122.4 (6)	C46C—C41C—C4C	121.5 (6)
C41A—C42A—C43A	123.3 (9)	C43C—C42C—C41C	119.7 (7)
C41A—C42A—H42A	118.4	C43C—C42C—H42C	120.2
C43A—C42A—H42A	118.4	C41C—C42C—H42C	120.2
C44A—C43A—C42A	120.1 (9)	C42C—C43C—C44C	120.6 (8)
C44A—C43A—H43A	119.9	C42C—C43C—H43C	119.7
C42A—C43A—H43A	119.9	C44C—C43C—H43C	119.7
C43A—C44A—C45A	117.7 (9)	C45C—C44C—C43C	118.9 (8)
C43A—C44A—H44A	121.2	C45C—C44C—H44C	120.5
C45A—C44A—H44A	121.2	C43C—C44C—H44C	120.5
C44A—C45A—C46A	121.3 (9)	C46C—C45C—C44C	120.5 (8)
C44A—C45A—H45A	119.3	C46C—C45C—H45C	119.7
C46A—C45A—H45A	119.3	C44C—C45C—H45C	119.7
C41A—C46A—C45A	119.0 (8)	C45C—C46C—C41C	124.1 (8)
C41A—C46A—H46A	120.5	C45C—C46C—H46C	118.0
C45A—C46A—H46A	120.5	C41C—C46C—H46C	118.0
C4A—C5A—N1A	107.9 (6)	C4C—C5C—N1C	107.5 (6)
C4A—C5A—H5A	126.1	C4C—C5C—H5C	126.3
N1A—C5A—H5A	126.1	N1C—C5C—H5C	126.3
C2B—N1B—C5B	111.1 (5)	C2D—N1D—C5D	110.1 (6)
C2B—N1B—H1B	124.5	C2D—N1D—H1D	125.0
C5B—N1B—H1B	124.5	C5D—N1D—H1D	125.0
N3B—C2B—N1B	104.8 (6)	N1D—C2D—N3D	105.5 (6)
N3B—C2B—S2B	129.0 (5)	N1D—C2D—S2D	126.5 (5)
N1B—C2B—S2B	126.2 (5)	N3D—C2D—S2D	128.0 (5)
C2B—N3B—C4B	111.9 (5)	C2D—N3D—C4D	111.1 (5)
C2B—N3B—H3B	124.0	C2D—N3D—H3D	124.5
C4B—N3B—H3B	124.0	C4D—N3D—H3D	124.5
C5B—C4B—N3B	105.1 (5)	C5D—C4D—N3D	106.5 (6)
C5B—C4B—C41B	130.8 (6)	C5D—C4D—C41D	128.3 (6)
N3B—C4B—C41B	124.2 (6)	N3D—C4D—C41D	125.2 (6)
C42B—C41B—C46B	119.4 (7)	C42D—C41D—C46D	115.1 (7)
C42B—C41B—C4B	121.9 (7)	C42D—C41D—C4D	123.5 (7)
C46B—C41B—C4B	118.7 (6)	C46D—C41D—C4D	121.2 (6)
C41B—C42B—C43B	117.5 (9)	C43D—C42D—C41D	123.8 (8)
C41B—C42B—H42B	121.2	C43D—C42D—H42D	118.1
C43B—C42B—H42B	121.2	C41D—C42D—H42D	118.1
C44B—C43B—C42B	124.9 (9)	C42D—C43D—C44D	117.1 (8)
C44B—C43B—H43B	117.6	C42D—C43D—H43D	121.5
C42B—C43B—H43B	117.6	C44D—C43D—H43D	121.5
C43B—C44B—C45B	116.0 (8)	C45D—C44D—C43D	121.2 (8)
C43B—C44B—H44B	122.0	C45D—C44D—H44D	119.4
C45B—C44B—H44B	122.0	C43D—C44D—H44D	119.4
C44B—C45B—C46B	122.7 (9)	C44D—C45D—C46D	119.9 (9)
C44B—C45B—H45B	118.6	C44D—C45D—H45D	120.0
C46B—C45B—H45B	118.6	C46D—C45D—H45D	120.0
C41B—C46B—C45B	119.5 (8)	C45D—C46D—C41D	122.7 (7)
C41B—C46B—H46B	120.3	C45D—C46D—H46D	118.6
C45B—C46B—H46B	120.3	C41D—C46D—H46D	118.6
C4B—C5B—N1B	107.2 (6)	C4D—C5D—N1D	106.9 (6)
C4B—C5B—H5B	126.4	C4D—C5D—H5D	126.6
N1B—C5B—H5B	126.4	N1D—C5D—H5D	126.6

### Hydrogen-bond geometry (Å, º)

**Table:**

*D*—H···*A*	*D*—H	H···*A*	*D*···*A*	*D*—H···*A*
N1*A*—H1*A*···S2*B*	0.86	2.44	3.274 (6)	163
N3*A*—H3*A*···S2*B*^i^	0.86	2.50	3.350 (5)	172
C42*A*—H42*A*···S2*B*^i^	0.93	2.85	3.723 (9)	156
N1*B*—H1*B*···S2*A*^ii^	0.86	2.46	3.290 (5)	163
N3*B*—H3*B*···S2*A*	0.86	2.48	3.343 (5)	176
C42*B*—H42*B*···S2*A*	0.93	2.79	3.686 (9)	161
N1*C*—H1*C*···S2*D*	0.86	2.45	3.288 (5)	166
N3*C*—H3*C*···S2*D*^ii^	0.86	2.50	3.348 (5)	172
C42*C*—H42*C*···S2*D*^ii^	0.93	2.89	3.741 (8)	153
N1*D*—H1*D*···S2*C*^i^	0.86	2.43	3.271 (6)	166
N3*D*—H3*D*···S2*C*	0.86	2.50	3.353 (6)	172
C42*D*—H42*D*···S2*C*	0.93	2.85	3.766 (9)	169

Symmetry codes: (i) *x*+1/2, *y*+1/2, *z*; (ii) *x*−1/2, *y*−1/2, *z*.
